# What’s in a Name?

**Published:** 1875-08

**Authors:** 


WHAT'S IN A NAME?—A correspondent of the *Dublin Medical Press* gets off the following very good satire on the names of the Dublin medical men. He says:

On looking over the names of our Dublin medical men, it has occurred to me that much convenience would result from each devoting himself to that branch of his profession indicated by his name. Thus I would place the lunatic asylum under the charge of Dr. Madden, the more violent cases being attended to by Dr. More Madden. I think that Drs. Boyes and Birch might fairly be deputed to look after the weaknesses of young persons; while Dr. Luther would be at home in charge of the Adelaide Hospital. The Lying-in Hospitals fall naturally to Drs. Bredin and Kidd; while hysteric affections should be treated by Drs. Cryan, Smyla and Laffan. Diseases of the bladder might be left to Dr. Stoney; while for baldness I do not know any more suitable advisers than Drs. Hare and Head. All matters relating to fees should be referred to Dr. Price; while attendance should be regulated by Dr. Daly. For lameness I would consult Drs. Walker and Foot; for gunshot wounds, Dr. Gunn; for operative surgery, undoubtedly surgeons Steele, Butcher and Gore would be selected. For skin diseases I would call in Dr. Peele; while questions of food might be left to Drs. Fry, Boyle, Cooke, Rice and Porter.—*Canada Record.*

